# Editorial: Children's Development During Social Transition

**DOI:** 10.3389/fpubh.2021.794444

**Published:** 2021-11-10

**Authors:** Stevo Popovic, Gregor Starc, Maroje Soric, Juel Jarani

**Affiliations:** ^1^Faculty for Sport and Physical Education, University of Montenegro, Niksic, Montenegro; ^2^Western Balkan Sport Innovation Lab, Podgorica, Montenegro; ^3^Faculty of Sport, University of Ljubljana, Ljubljana, Slovenia; ^4^Faculty of Kinesiology, University of Zagreb, Zagreb, Croatia; ^5^Faculty of Movement Sciences, Sports University of Tirana, Tirana, Albania

**Keywords:** physical activity, exercise, physical fitness, kids, growth

## Introduction

Adaptability is one of the key traits that enabled human kind to survive numerous environmental challenges throughout its history and spread throughout all the continents. Initially the main drivers of adaptations were natural factors such as climate and availability of food but with the settling down, society started to emerge and started adding its demands also for social adaptation. Culture and technology became the main sources of adaptational pressure which strongly intensified in modern times with industrial revolution and especially with communication revolution. The pace of lifestyle changes from the mid-twentieth century onwards has been exponentially increasing and profoundly changing the relationship between humans and our technology. Our natural environment has merged with technological environment and the constant technological revolution is forcing people to adapt to our own inventions. In adults, such environmental pressures induce mostly changes in our behavior, while in children, the environmental pressure can irreversibly alter the mode and pace of their development. Technological revolution is simultaneously intensifying also the socio-economic changes which are further altering children's environment. This environment is becoming extremely unstable and is characterized by constant transition.

When John Locke developed his “Tabula Rasa” theory as early as the seventeenth century in which he explained that “children come into the world with an empty mind, and that knowledge and learning is received through experience and converted to understanding through reasoning,” he couldn't have imagined the multiplication of factors that influence the development of contemporary children. In this regard children's development has become extremely complex because the biological laws are being tested by various changes of economic, cultural, technological, ecological, and institutional developments on different scales (1). Facing the challenges of children's development in social transition, this Research Topic was created with intention to help the upbringing of strong children rather than to repair broken adults.

The purpose of this Research Topic was to gather the latest knowledge in the field of children's development during social transition. The 12 studies that emerged as the output of this special issue have advanced the field in several ways.

First, some very interesting findings were reached in this Research Topic related to the monitoring of trends in children's development. The geographical variation in physical fitness among Chinese children and adolescents was explored by Dong et al. The authors concluded that the physical fitness showed an improved trend from 2005 to 2014 and that region-specific interventions with priority policies could be useful to sustainably reduce geographical inequalities in fitness of Chinese children, especially in the western provinces. Additionally, two systematic reviews analyzed trends in physical fitness among school-aged children and adolescents over the last 50 years (Masanovic et al.), and identified available field-based health-related physical fitness tests in children and adolescents that can be used in school settings (Marques et al.). These studies detailed the complex trend in physical fitness among school-aged children and adolescents that differed across the globe, and highlighted a need for a consensus and standardization of physical fitness assessments in school environment.

Another important stream of work is reflected in the studies that analyzed: (1) trajectories in body height, body weight, BMI, and nutrition status of male adolescents from the capital city of Montenegro (Popovic et al.), and (2) relationship between national economic development and body mass index in Chinese children and adolescents (Bu et al.). These studies help to characterize secular trends in one of the tallest nations in the world from the beginning of the twentieth century—Montenegro—which has specific body proportions, as well as the relationship between economic development and body mass index in Chinese children and adolescents during the economic transition. Both studies emphasized the importance of globalization and recognized its impact on children's development.

Next, one study in this special issue examined the influence of skeletal age and chronological age on pre-schoolers' physical fitness performance and found both to be related to skill-related, but not health-related fitness (Ke et al.). Another investigation examined dietary patterns and weight status of 6–9 year-old children and identified irregular breakfast consumption and sugar-sweetened bevarages intake as the most important risk factors for obesity (Bozic et al.). The timeliness of this topic is particularly reflected in two studies that have investigated the impact of COVID-19 pandemic on children's development. Vuković et al. investigated the children's daily routine response to COVID-19 emergency measures, while López-Bueno et al. evaluated health-related behaviors among school-aged children and adolescents during the COVID-19 confinement. These studies show support for a marked decrease in children's physical activity, but also suggest that more active children showed higher mental resilience during this public health crisis. Similarly, Ahad et al. in their opinion paper warned about possible spill over trends of child labor during the COVID-19 crisis.

Lastly, one of the studies from this special issue (Chao and Cheng) found that an educational course can be effective in improving the teenagers' understanding of the impact of adolescence on attitudes toward life, sex, gender equality, and mental health, while another study (Mieziene et al.) explored the direct and indirect relationships within the extended trans contextual model for moderate-to-vigorous physical activity in Physical Education and during leisure time, and demonstrated that the main goal of physical education of enhancing physical activity not only in school but also outside of school, is working.

## Conclusion

Children's development is determined by genetic, but also by social factors, which shape and determine their everyday life. However, environmental factors in the contemporary world are becoming less stable and less predictable, especially in the conditions of rapid social changes such as socio-political, economic, and public health-related events that have been profoundly affecting the lifestyles of children in the last three decades. In this period, we witnessed the arrival of capitalist consumerism in ex-socialist European countries, rapid worldwide urbanization of previously rural societies, the global financial crisis of 2007–08, and the global COVID-19 pandemic of 2020, which all triggered profound and sudden transformations of society. Children had to adapt to each new situation, and studies compiled in this special issue indicate that these newly adapted social practices most often displaced otherwise habitual physical activity with sedentariness, causing the myriad of increased risks to normal somatic and motor development of children. Therefore, now, more than ever, understanding and developing strategies to promote physical activity behavior and to improve children's fitness levels are essential. These strategies can be developed in the school setting or in different contexts. To this end, this Research Topic leveraged high-quality research studying changes in children's development during social transition to offer guidance to policy makers around the globe on how to alleviate the consequences of abrupt societal changes.

## Author Contributions

SP drafted the editorial. GS, MS, and JJ revised and approved the final version. All authors contributed to the article and approved the submitted version.

## Conflict of Interest

The authors declare that the research was conducted in the absence of any commercial or financial relationships that could be construed as a potential conflict of interest.

## Publisher's Note

All claims expressed in this article are solely those of the authors and do not necessarily represent those of their affiliated organizations, or those of the publisher, the editors and the reviewers. Any product that may be evaluated in this article, or claim that may be made by its manufacturer, is not guaranteed or endorsed by the publisher.
